# Duodenal-jejunal bypass normalizes pancreatic islet proliferation rate and function but not hepatic steatosis in hypothalamic obese rats

**DOI:** 10.1590/1414-431X20175858

**Published:** 2017-03-30

**Authors:** K.R. Cantelli, G.M. Soares, R.A. Ribeiro, S.L. Balbo, C. Lubaczeuski, A.C. Boschero, A.C.F. Araújo, M.L. Bonfleur

**Affiliations:** 1 Laboratório de Fisiologia Endócrina e Metabolismo, Centro de Ciências Biológicas e da Saúde, Universidade Estadual do Oeste do Paraná, Cascavel, PR, Brasil; 2 Universidade Federal do Rio de Janeiro, Macaé, RJ, Brasil; 3 Laboratório de Pâncreas Endócrino e Metabolismo, Departamento de Biologia Estrutural e Funcional Instituto de Biologia, Universidade Estadual de Campinas, Campinas, SP, Brasil; 4 Centro de Ciências Médicas e Farmacêuticas, Universidade Estadual do Oeste do Paraná, Cascavel, PR, Brasil

**Keywords:** Duodenal-jejunal bypass, Hepatic fatty acid metabolism, Hypothalamic obesity, Ki67, Insulin secretion

## Abstract

Modifications in life-style and/or pharmacotherapies contribute to weight loss and ameliorate the metabolic profile of diet-induced obese humans and rodents. Since these strategies fail to treat hypothalamic obesity, we have assessed the possible mechanisms by which duodenal-jejunal bypass (DJB) surgery regulates hepatic lipid metabolism and the morphophysiology of pancreatic islets, in hypothalamic obese (HyO) rats. During the first 5 days of life, male Wistar rats received subcutaneous injections of monosodium glutamate (4 g/kg body weight, HyO group), or saline (CTL). At 90 days of age, HyO rats were randomly subjected to DJB (HyO DJB group) or sham surgery (HyO Sham group). HyO Sham rats were morbidly obese, insulin resistant, hypertriglyceridemic and displayed higher serum concentrations of non-esterified fatty acids (NEFA) and hepatic triglyceride (TG). These effects were associated with higher expressions of the lipogenic genes and fatty acid synthase (FASN) protein content in the liver. Furthermore, hepatic genes involved in β-oxidation and TG export were down-regulated in HyO rats. In addition, these rats exhibited hyperinsulinemia, β-cell hypersecretion, a higher percentage of islets and β-cell area/pancreas section, and enhanced nuclear content of Ki67 protein in islet-cells. At 2 months after DJB surgery, serum concentrations of TG and NEFA, but not hepatic TG accumulation and gene and protein expressions, were normalized in HyO rats. Insulin release and Ki67 positive cells were also normalized in HyO DJB islets. In conclusion, DJB decreased islet-cell proliferation, normalized insulinemia, and ameliorated insulin sensitivity and plasma lipid profile, independently of changes in hepatic metabolism.

## Introduction

Obesity predisposes to insulin resistance and contributes to the pathogenesis of type 2 diabetes (T2D) (1). Insulin resistance also disrupts whole body lipid metabolism, leading to hypertriglyceridemia and accumulation of triglycerides (TG) in the liver, promoting nonalcoholic fatty liver disease (NAFLD) (2). Insulin resistance is counteracted by an increase in insulin secretion, due to morphological and functional alterations in pancreatic β-cells. However, when β-cell compensatory modifications cannot be sustained, T2D is established (3,4).

The hypothalamus is a key regulator of body mass, controlling food intake, energy expenditure, and body fat stores. Hypothalamic damage, promoted by genetic defects, radiotherapy or the resection of a brain tumor can lead to neuroendocrine dysfunctions resulting in morbid obesity (5). Hypothalamic obese (HyO) patients display hyperinsulinemia, insulin resistance, hypertriglyceridemia, hyperleptinemia and NAFLD (6,7). Life-style modifications, physical activity and/or pharmacotherapy are used for weight loss in genetically and diet-induced obesity (8), but often fail in HyO patients (9). An alternative for these patients may be bariatric surgery, but the effects of this procedure in HyO individuals are controversial (5,9,10).

Duodenal-jejunal bypass (DJB) surgery seems to be a good strategy to improve glucose homeostasis and NAFLD in obese rodents, induced by hyper-caloric diet or genetic alterations (11–14). However, information about the effects of DJB upon hypothalamic obesity is scarce, possibly due to a lack of experimental models that mimic the features of this syndrome. The treatment of neonatal rodents with monosodium glutamate (MSG) leads to hypothalamic lesions that promote neuroendocrine dysfunctions and metabolic disruptions, similar to those observed in HyO humans. MSG-obese rats presented morbid obesity, NAFLD, glucose intolerance, insulin resistance, hyperinsulinemia, hyperleptinemia, and pancreatic islet hyperfunction (15–17). DJB surgery in MSG-treated rats does not decrease adiposity and hepatic steatosis, but ameliorates body glucose control and hepatic insulin action (18). Thus, we aimed to better understand the mechanism of action by which DJB surgery regulates hepatic lipid metabolism and pancreatic islet morphophysiology in HyO rats.

## Material and Methods

### Induction of hypothalamic obesity and DJB surgery protocol

Male newborn Wistar rats received a daily subcutaneous injection of MSG [4 g/kg body weight (BW), HyO group], or hyperosmotic saline (1.25 g/kg BW, CTL group, n=19) during the first 5 days of life. All rats were maintained on a 12-h light/dark cycle (lights on from 6:00 am to 6:00 pm) and controlled temperature (22±1°C), with free access to standard rodent chow (Biobase^®^, Brazil) and water.

At 90 days of age, HyO rats were randomly submitted to DJB (HyO DJB group, n=18) or sham surgery (HyO Sham, n=12). Preoperative procedures were performed as previously described by Meguid et al. (19). Rats were deprived of food for 12 h and were anesthetized with isoflurane (Isoforine^®^, Brazil). For DJB surgery, HyO rats were laparotomized and a postpyloric transection was made to separate the stomach from the duodenum. The reconstruction of the intestinal passage was performed in the terminolateral duodenojejunostomy 5 cm aboral of the flexura duodenojejunalis. In HyO Sham rats, after the laparotomy the stomach, duodenum and intestines were massaged, and the incision was closed. Six HyO DJB rats died from post-surgical complications (mortality rate 33%). All experiments were approved by the Universidade Estadual do Oeste do Paraná Committee on Ethics in Animal Experimentation.

### Evaluation of obesity and biochemical parameters

BW was measured monthly during the experimental period. The Lee index was calculated as follows: BW (g)^1/3^/nasoanal length (cm)×1000 (20). Blood was obtained by a cut in the tail tip from 8-h fasted rats, and glucose was measured using a glucose analyzer (Abbott^®^, Optium Xceed, USA). Subsequently, the rats were euthanized by decapitation and total blood was collected to obtain the serum, which was used to measure TG, total cholesterol (CHOL) and non-esterified fatty acids (NEFA) with standard commercial kits, according to the manufacturers’ instructions (Laborclin^®^, Bioliquid, Brazil and Wako^®^, Germany, respectively). Serum insulin was measured by radioimmunoassay.

### Liver TG content and HOMA-IR

Fragments from the liver (right middle lobe; RML) were collected and lipids were extracted by the Folch’s method (21). The extract was evaporated and then diluted in isopropanol, and TG was measured as described above. Tissue insulin sensitivity was evaluated by the previously validated homeostasis model assessment index of insulin resistance (HOMA-IR) (22).

### Static insulin secretion

Pancreatic islets were isolated by the collagenase digestion of pancreases. For static incubation, groups of four islets from each group were first incubated for 30 min in Krebs-Ringer bicarbonate (KRB) solution containing: 115 mM NaCl, 5 mM KCl, 2.56 mM CaCl_2_, 1 mM MgCl_2_, 10 mM NaHCO_3_, 15 mM HEPES, supplemented with 5.6 mM glucose, 3 g BSA/L, and equilibrated with a mixture of 95% O_2_/5% CO_2_ to give a pH of 7.4. This medium was then replaced with fresh KRB buffer and the islets were incubated for a further 1 h in the presence of 5.6 or 8.3 mM glucose. At the end of the incubation period, aliquots of the supernatant were collected and maintained at –20°C for posterior insulin measurement by radioimmunoassay.

### Pancreas morphometry and immunohistochemistry

The pancreas from all groups of rats was removed, weighed and fixed for 24 h in 4% formaldehyde solution (Sigma Aldrich Chemicals, USA). The tissue was then embedded in Paraplast^®^ (Sigma Aldrich Chemicals). From each pancreas, five consecutive 7-μm serial sections were selected, and after an interval of 140 μm in thickness, five more consecutive sections were obtained. Three sections were randomly selected for insulin, two sections for glucagon, and one for the Ki67 immunoperoxidase reaction. For immunohistochemistry, Paraplast^®^ was removed, the sections were rehydrated and washed with 0.05 M tris-saline buffer (TBS) pH 7.4, and incubated with TBS containing 0.3% H_2_O_2_ for endogenous peroxidase activity blockade and permeabilized for 1 h with TTBS (0.1% Tween 20 and 5 g/% of fat free milk in TBS). The sections were incubated with a polyclonal guinea pig anti-insulin (1:150; Dako North America, Inc., USA), or rabbit anti-glucagon (1:50; Dako North America, Inc.), or rabbit monoclonal anti Ki67 (1:20; Spring Bioscience, USA) antibody at 4°C overnight. Subsequently, the sections were incubated with rabbit anti-guinea pig IgG or goat anti-rabbit conjugated antibody with HRP for 1 h and 30 min. The positive insulin, glucagon or Ki67 cells were detected with diaminobenzidine (DAB; Sigma-Aldrich Chemicals) solution (10% DAB and 0.2% H_2_O_2_ in TBS). Finally, the sections were quickly stained with Ehrlich’s hematoxylin and mounted for microscopic observation. All islets present in the sections were covered systematically by capturing images with a digital camera coupled to a microscope (Olympus DP71; Olympus BX60, Japan). Pancreatic islet, β-cell and α-cell areas were measured using the Image-Pro-Plus Media, Cybernetics Software (USA). The islet, β-cell and α-cell masses were calculated by the total islets, β-cell and α-cell areas (% pancreas area) multiplied by pancreas weight (mg) (17). The proliferation of islet cells is reported as the percentage of nuclei in islets stained for Ki67 protein.

### Isolation of RNA and qPCR

Total RNA from the RML of liver was isolated using the PuriLink^®^ RNA mini kit (Life Technologies, USA). Quantification of mRNAs were performed using the 7500 Fast Real-Time PCR System (Applied Biosystems, USA), and the expression concentration of each amplified gene was normalized to that of the *glyceraldehyde 3-phosphate dehydrogenase* (*GAPDH*) gene. The absolute amount of gene expression was calculated by the use of standard curves (10^8^-10^3^ copies/2 μL DNA molecules), produced from the gene amplification products on 2% agarose gels. Primer sequences used for rat gene are described in Supplementary Table S1.

### Western blot

For protein expression experiments, fragments of RML of liver were solubilized in homogenization buffer (100 mM Tris pH 7.5, 10 mM sodium pyrophosphate, 100 mM sodium fluoride, 10 mM EDTA, 10 mM sodium vanadate, 2 mM PMSF and 1% Triton-X 100) at 4°C using a Polytron MA 102/mini homogenizer (Marconi^®^, Brazil). The protein concentration in the supernatants was assayed using a commercial Bradford reagent (Bio-Agency Lab., Brazil) and BSA for the standard curve. For SDS gel electrophoresis, the protein samples were homogenized with a loading buffer containing dithiothreitol and heated at 95°C for 5 min. Subsequently, the proteins were separated by electrophoresis (100 µg protein/lane in 6.5% gels) and transferred to nitrocellulose membranes. The membranes were blotted with specific primary antibodies against ACC, phospho-pACC^Ser79^ (Cell Signaling Technology, USA), FASN (Santa Cruz, USA), and CPT-1a (Abcam, Inc., USA). Visualization of specific protein bands was carried out by incubating the membranes with secondary antibodies, and images were captured using a PhotoDocumentor (L-Pix Chemi Express, Loccus Biotecnology, Brazil). LabImage 1D software was used to analyze the density of the bands (Loccus Biotecnology, SP, BRA). The α-tubulin protein (Sigma-Aldrich Chemicals) was used as a control of protein expression.

### Statistical analysis

Results are reported as means±SEM. Data were analyzed using one-way ANOVA followed by the Tukey post-test (P<0.05) with the GraphPad Prism^®^ Software version 5.00 (USA).

## Results

### General rat parameters

**Figure 1:**
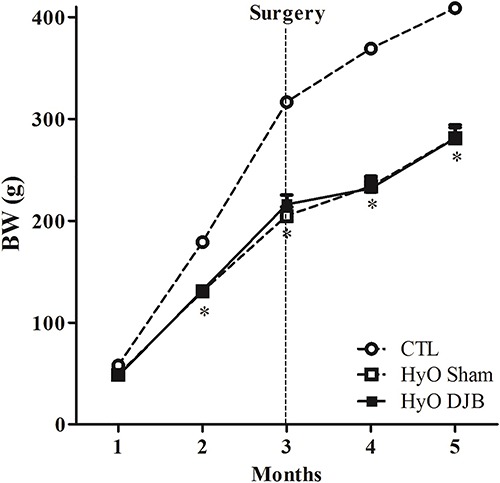
Body weight (BW) in control (CTL), hypothalamic obese (HyO) Sham and HyO with duodenal-jejunal bypass (DJB) rats, recorded during 5 months. Data are reported as means±SEM (n=13–16). *P<0.05, HyO Sham and HyO DJB groups compared to CTL (one-way ANOVA followed by the Tukey post-test).

HyO rats showed a significantly lower BW at 2 months of age; this weight remained lower compared to CTL rats until the end of the experimental period (P<0.05). DJB surgery did not modify BW in HyO DJB rats compared with HyO Sham rats (Figure 1).

At the end of the experimental period, the final BW and nasoanal length were lower in the HyO Sham rats compared with CTL (both P<0.001). However, the HyO Sham group displayed an enhanced Lee index and increases of 63 and 92% in the retroperitoneal and perigonadal fat stores compared with CTL rats (P<0.01, P<0.001, and P<0.001, respectively). At 2 months after DJB surgery, all of these parameters were similar to those found in the HyO Sham group (Table 1).

**Table 1:**
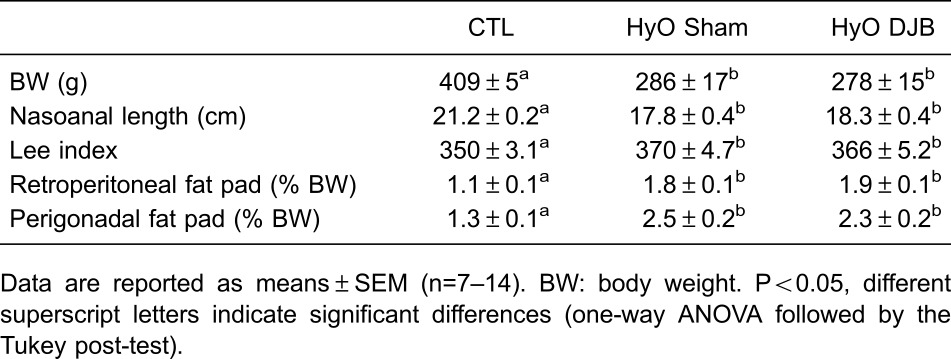
Obesity parameters evaluated in control (CTL), hypothalamic obese (HyO) Sham and HyO with duodenal-jejunal bypass (DJB) rats.

### Serum and hepatic lipid profiles

We investigated the effects of HyO and DJB on body lipid homeostasis. HyO Sham rats presented higher NEFA serum concentrations, hypertriglyceridemia and higher TG content in the liver (P<0.001). At 2 months after surgery, serum NEFA and TG were reduced by 42 and 26%, respectively, in HyO DJB rats compared with HyO Sham rats (P<0.001 and P<0.01). However, the hepatic TG content in HyO DJB rats was similar to that of the HyO Sham rats and higher than that of the CTL group. No modifications in total serum CHOL values were observed in the experimental groups (Table 2).

**Table 2:**
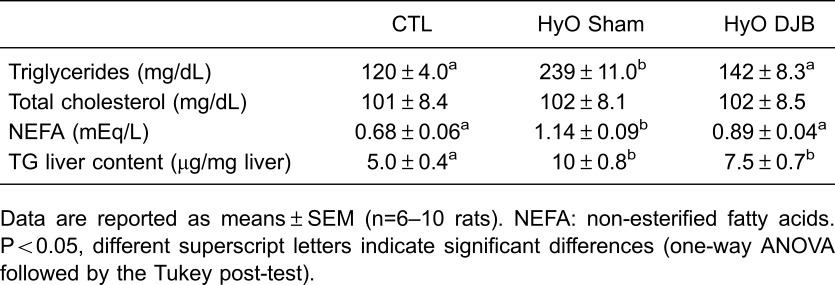
Serum lipid parameters and triglycerides (TG) liver content in control (CTL), hypothalamic obese (HyO) Sham and HyO with duodenal-jejunal bypass (DJB) rats.

**Figure 2:**
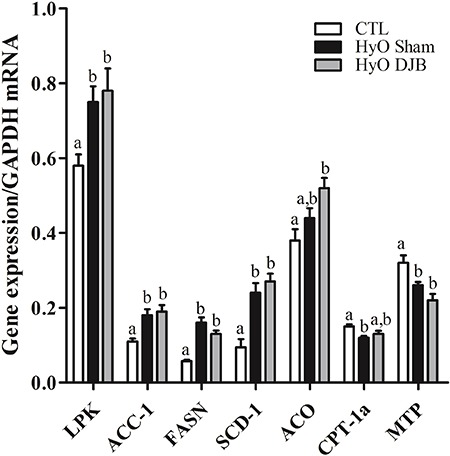
*LPK*, *ACC-1*, *FASN*, *SCD-1*, *ACO*, *CPT-1a* and *MTP* mRNA content in the livers of control (CTL), hypothalamic obese (HyO) Sham and HyO with duodenal-jejunal bypass (DJB) rats. Data are reported as means±SEM (n=5–8). P<0.05, different letters indicate significant differences (one-way ANOVA followed by the Tukey post-test).

**Figure 3:**
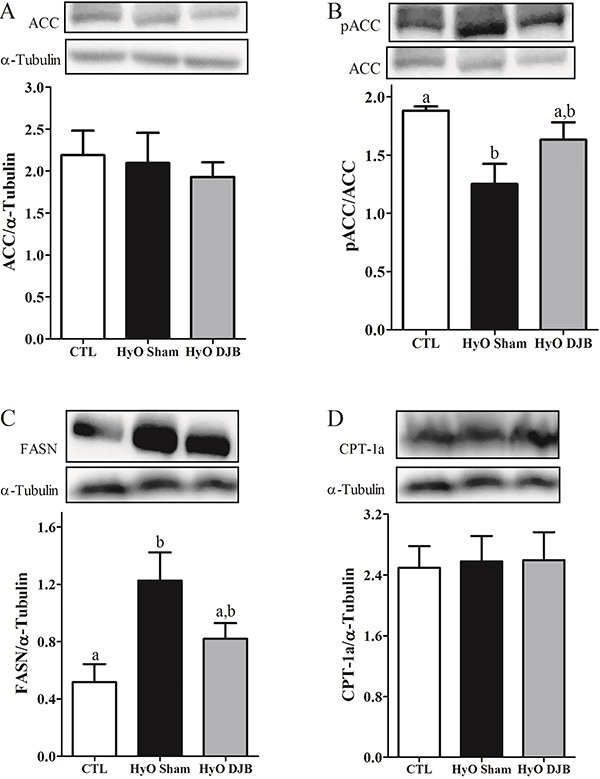
Hepatic protein expressions of ACC (*A*), pACC^Ser79^/ACC (*B*), FASN (*C*) and CPT-1a (*D*) in control (CTL), hypothalamic obese (HyO) Sham and HyO with duodenal-jejunal bypass (DJB) rats. Data are reported as means±SEM (n=5–8 rats). P<0.05, different letters indicate significant differences (one-way ANOVA followed by the Tukey post-test).

To better characterize hepatic lipid metabolism in HyO rats, the expression of several genes and proteins involved in *de novo* lipogenesis and fatty acid (FA) β-oxidation were investigated. When compared to the CTL rats, HyO Sham rats showed a higher expression of the hepatic lipogenic mRNAs, *LPK*, *ACC-1*, *FASN* and *SCD-1* (P<0.05, P<0.05, P<0.001, and P<0.01, respectively), but reduced *CPT-1a* and *MTP* gene expressions, key factors involved in β-oxidation and in assembling of the TG-rich ApoB-containing lipoproteins, respectively (P<0.05 and P<0.001; Figure 2). However, only the hepatic protein content of FASN, an enzyme that catalyzes the synthesis of long-chain FA from acetyl-CoA and malonyl-CoA, was significantly higher in the liver of HyO Sham rats compared with CTL animals (P<0.05; Figure 3C). In addition, pACC/ACC protein expression was significantly reduced in the liver of HyO Sham rats (P<0.04: Figure 3B), indicating an increased ACC activity and, therefore, higher FA synthesis. DJB surgery failed to normalize hepatic gene and protein expressions of the enzymes involved in *de novo* lipogenesis and β-oxidation (Figures 2 and 3).

### Glucose homeostasis

**Figure 4:**
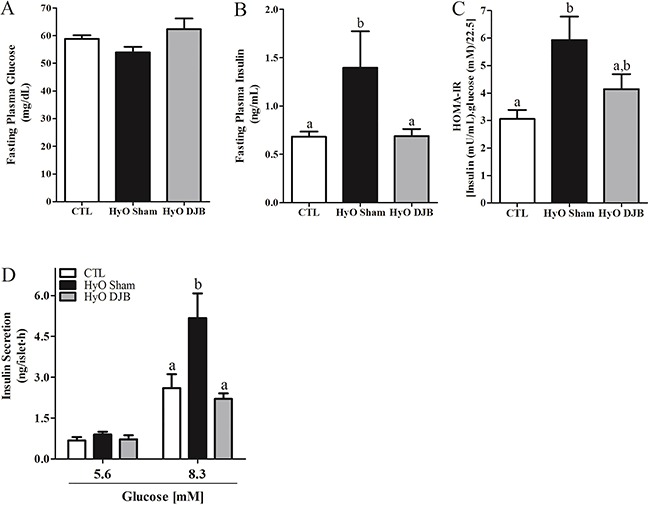
Means±SEM (n=8-14) of *A*, serum glucose concentration, *B*, insulin concentration, and *C,* insulin sensitivity measured by the HOMA-IR in fasted control (CTL), hypothalamic obese (HyO) Sham and HyO with duodenal-jejunal bypass (DJB) rats. *D*, Glucose-induced insulin secretion in islets isolated from CTL, HyO Sham and HyO DJB rats. Groups of 4 islets were incubated for 1 h in the presence of 5.6 or 8.3 mM glucose. Data are reported as means±SEM obtained from 8–12 groups of islets in two independent experiments with 4 rats per group. P<0.05, different letters indicate significant differences (one-way ANOVA followed by the Tukey post-test).

HyO Sham rats presented normal fasting glycemia, but higher insulinemia (P<0.02), indicating an impaired insulin action in peripheral tissues, as indicated by the higher HOMA-IR index in HyO Sham rats compared with CTL (P<0.003). DJB surgery reduced the insulinemia to values similar to those observed for the CTL; however, the HyO DJB rats showed only a partial reduction in HOMA-IR (Figure 4A-C).

The higher insulinemia, observed in the HyO Sham group, was associated with enhanced pancreatic β-cell responsiveness to glucose, since insulin secretion at 8.3 mM glucose in islets from HyO Sham rats was 1.9-fold greater, than in CTL islets (P<0.05). The reduction in insulinemia in HyO rats, observed at 2 months after DJB surgery, was accompanied by a decrease in insulin release, in response to 8.3 mM glucose in HyO DJB islets compared with HyO Sham rats (P<0.01; Figure 4D).

### Pancreatic islet morphology and morphometry

**Figure 5:**
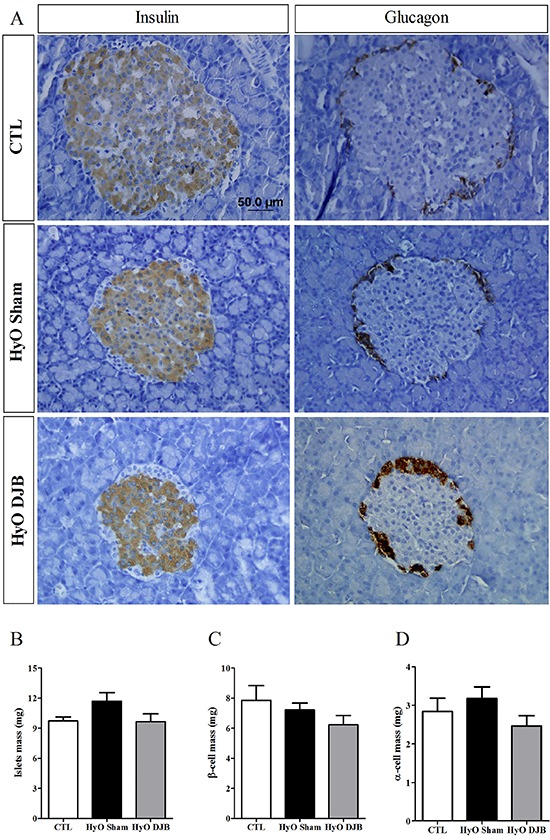
*A*, Representative images of pancreas sections stained for insulin or glucagon. *B*, Islet, *C*, β-cell masses, and *D*, α-cell masses in the CTL, hypothalamic obese (HyO) Sham and HyO with duodenal-jejunal bypass (DJB) pancreases. Data are reported as means±SEM (n=3–4). P<0.05, different letters indicate significant differences (one-way ANOVA followed by the Tukey post-test).

**Figure 6:**
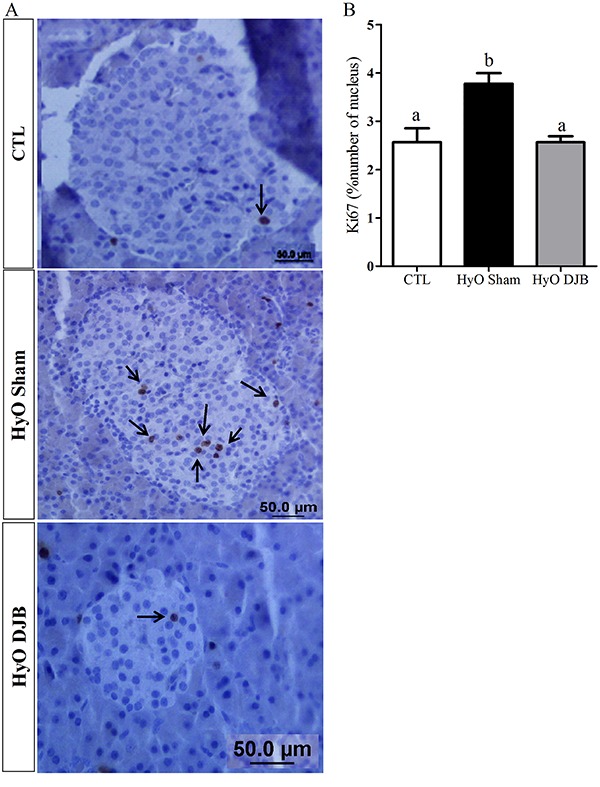
*A*, Representative images of pancreas sections stained for Ki67. *B*, Percent of Ki67 positive nucleus in the control (CTL), hypothalamic obese (HyO) Sham and HyO with duodenal-jejunal bypass (DJB) pancreases. Arrows represent the nucleus stained by Ki67. Data are reported as means±SEM (n=3–4). P<0.05, different letters indicate significant differences (one-way ANOVA followed by the Tukey post-test).

We analyzed whether the islet function modifications induced by DJB surgery in HyO rats were due to alterations in endocrine pancreas morphology. HyO Sham rats presented 33% lower pancreas relative weight per BW compared to the CTL rats (P<0.01). At 2 months after DJB surgery, HyO DJB rats displayed a 1.6-fold increase in pancreas relative weight compared with HyO Sham rats (P<0.01; Table 3). Histological analysis of the pancreas of CTL, HyO Sham and HyO DJB groups did not reveal any significant difference in the islet architecture. The islets from these groups were approximately spherical or oval in shape, displaying a typical β-cell arrangement within the islet core and α-cells at the periphery (Figure 5A). However, the size of the islets, as well as the areas of α- and β-cells, were smaller in HyO Sham rats than that observed in the CTL pancreas (both P<0.001). In contrast, the pancreas of HyO Sham pancreas presented a greater percentage of total islet and β-cell areas per pancreas section than the CTL pancreas (P<0.001; Table 3). However, islets, β-cell and α-cell masses did not differ between the HyO Sham and CTL groups (Figure 5B–D). In contrast, HyO Sham pancreatic islets presented a higher percentage of nuclei stained for Ki67 protein in comparison to CTL islets (P<0.001; Figure 6A and B). At 2 months after DJB surgery, HyO DJB pancreatic islets, β and α-cell areas did not differ from those of the HyO Sham group (Table 2), although significant reductions in the total percentage of islet and β-cell areas per pancreas section were evidenced in HyO DJB compared with the HyO Sham group (P<0.001; Table 3). In addition, HyO DJB pancreatic islets presented a reduced percentage of Ki67-positive nuclei compared to that of HyO sham rats (P<0.001; Figure 6B).

**Table 3:**
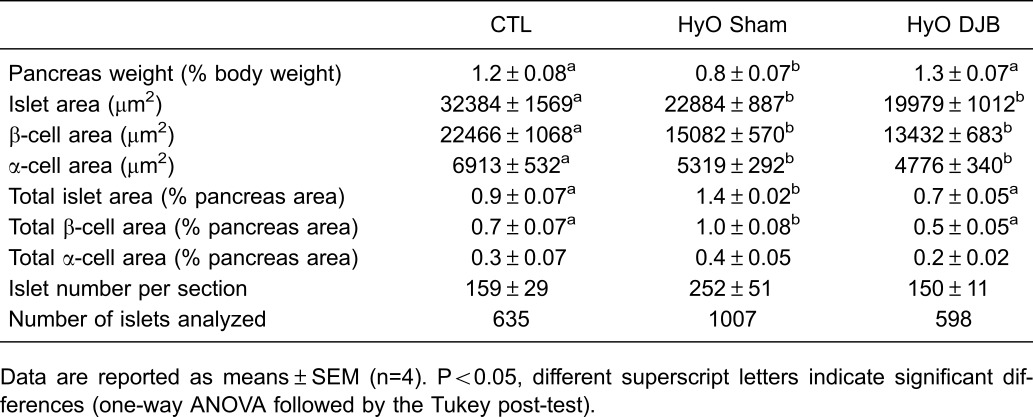
Morphometric analysis of the pancreas from control (CTL), hypothalamic obese (HyO) Sham and HyO with duodenal-jejunal bypass (DJB) rats.

## Discussion

Hypothalamic obesity is a major and unsolved problem in patients with hypothalamic lesions and has a major negative impact on survival and quality of life (23). We, herein, report data that provide evidence about the mechanism by which DJB surgery may regulate hepatic lipid metabolism and endocrine pancreatic morphofunction in HyO rats.

In accordance with our previous observations (15,16), and similar to observations in HyO patients (6,7), with the exception of the lower BW, HyO Sham rats were hypertriglyceridemic with higher serum NEFA and hepatic TG concentrations. These alterations may be associated with enhanced expression of *de novo* lipogenic genes (*LPK*, *ACC-1*, *FASN*, and *SCD-1*) and FASN protein, and ACC activation in the liver of HyO rats. In addition, the higher concentrations of NEFA in the serum of HyO Sham rats may enhance the accumulation of TG in the liver.

Circulating NEFAs are derived from three sources: the diet, *de novo* FA synthesis and circulating FA (24). The consumption of a high-fat or western diet leads to the development of hepatic steatosis (14,25). Conversely, it has been suggested that approximately 60% of liver fat is derived from circulating NEFA in individuals who eat a normal fat-containing diet (24). Since MSG hypothalamic lesions do not enhance food consumption (15), but lead to obesity and insulin resistance in skeletal muscle and adipose tissue (26), this effect contributes to increased plasma NEFA levels, due to increased lipid release from adipose tissue, which enhances the FA source to the liver.

Furthermore, reductions in hepatic FA β-oxidation also account for NAFLD (27). Although *CPT-1a* mRNA was down-regulated in the liver of HyO rats, the hepatic CPT-1a protein was not changed indicating that a post-transcriptional modification occurs in the liver of these rodents. However, *MTP* mRNA, which encodes a protein that participates in the TG transfer to nascent apolipoprotein B to form very low-density lipoproteins (28), was down-regulated in HyO Sham rats, indicating possible impairment in hepatic TG export.

Previous observations using diet-induced obesity in rodents demonstrated that the DJB intervention is beneficial against liver fat deposition. At 8 weeks after DJB surgery, high-fat diet rats treated with streptozotocin presented reductions in ACC and FASN protein levels, which lowered hepatic TG accumulation (25). In rats that consumed a western diet, DJB also decreased circulating and hepatic TG concentrations (14). Although HyO rats displayed normal circulating TG and NEFA concentrations, the expression of hepatic lipogenic genes was not decreased, nor was the expression of β-oxidation mRNAs improved at 2 months after DJB surgery. Furthermore, FASN protein and ACC activation were higher in HyO DJB rats. This FA hepatic metabolic profile may contribute to maintain the higher TG deposition in the liver of HyO DJB rats. Conversely, the normalization of NEFA serum concentrations in HyO DJB rats may be due to the partial restoration of insulin action, as demonstrated by HOMA-IR values in these rodents, suggesting that the improved insulin action in adipose tissue can contribute to decrease lipolysis and improve FA utilization by peripheral tissues. Therefore, these data indicate that HyO pathophysiology differs from other types of obesity. As such, it is plausible that therapeutic strategies frequently used against nutrition-related obesity do not provide full metabolic benefits in HyO patients.

In contrast, HyO rats were normoglycemic, despite the presence of a severe insulin resistance. Normoglycemia in these rats was supported by hyperinsulinemia, provided by insulin hypersecretion from the pancreatic β-cells. Similar features have been observed in HyO patients (6). Hyperinsulinemia in hypothalamic lesions is frequently associated with the disruption of the autonomic nervous system (ANS) in HyO humans and experimental rodents (29,30). The ANS dysfunction is characterized by a lower inhibitory sympathetic tone, associated with an augmented vagal parasympathetic signaling to the pancreatic β-cells. Furthermore, higher parasympathetic activity enhances β-cell mass and secretion under normal and pathological conditions (31,32). Accordingly, we recently demonstrated that HyO pancreatic islets present a higher β-cell number per islet, indicating that compensatory morphofunction alterations in the HyO pancreas are accompanied by enhanced islet-cell replication, induced by vagal hypertonia, since truncal vagotomy in HyO rats normalized β-cell amount per islet (17). In addition, we observed a higher number of Ki67-positive cells in the HyO endocrine pancreas, a marker of islet-cell proliferation, which accounted for the insulin hypersecretion from HyO islets. C57Bl/6 mice, submitted to a high-fat diet, are reported to show an expansion of their β-cell mass due to an increased β-cell proliferation rate during the first week of the diet and before the appearance of insulin resistance (4). This early β-cell replication may occur due to increased parasympathetic activity, since the vagus nerve controls cellular proliferation in normal and pathological conditions, as obesity (31,33,34).

DJB surgery, independent of changes in body adiposity, seems to be a good strategy to improve glucose homeostasis in pre-diabetic and diabetic experimental rodents and patients (13,18). However, the effects of DJB intervention upon the morphofunction of the endocrine pancreas are largely unknown. At 28 days after DJB surgery, high-fat diet mice did not present modifications in β-cell mass or β-cell proliferation (11). DJB surgery increased the β-cell area and reduced islet fibrosis after 12 months, in non-obese diabetic Goto-Kakizaki (GK) rats (12). Our study is the first to demonstrate that DJB normalizes islet-cell proliferation rate in HyO DJB rats. This effect may contribute to the normalization of islet and β-cell percentage per pancreas section in the HyO pancreas, contributing to normalize the β-cell secretory capacity. In addition, the improvement in hyperinsulinemia and insulin sensitivity in HyO DJB rats (18), may also be involved in such process.

The modifications in body glucose control and β-cell function that occur following bariatric surgery are frequently associated with the secretion of gut hormones such as glucagon-like peptide (GLP)-1 and glucose-dependent insulinotropic polypeptide (GIP) (12,35). These hormones potentiate glucose-induced insulin release (the so-called incretin effect) and regulate islet-cell survival (36). However, in GK or in Zucker rats, DJB surgery was not accompanied by an increase in GLP-1 and GIP plasma concentrations (37,38). As such, our results suggest that the benefits of bariatric surgeries upon endocrine pancreatic morphofunction may be linked to alterations in ANS function. A reduced vagal innervation close to the proximal and distal stomach of high-fat diet mice, submitted to Roux-en-Y gastric bypass (RYGB), was observed, without alterations in the density of vagal fiber innervation to the pancreas (39). In accordance, lean Sprague-Dawley rats, submitted to RYGB, presented reduced activation of afferent and efferent fibers of the gastric vagal branches, which disconnects the vagal signaling from the stomach to the hindbrain (40).

In summary, our study is the first to show that, at 2 months after DJB, HyO rats did not reduce hepatic TG accumulation due to the higher gene and protein expression of *de novo* lipogenic enzymes, together with reduced MTP mRNA, which probably impairs lipopoliprotein assembly. However, DJB presented benefits on endocrine pancreatic morphology in HyO rats, decreasing islet-cell hyperplasia by reducing the cellular proliferation rate, which contributes to normalize insulin release, insulinemia and, partly, the action of insulin.

## Supplementary Material

Click here to view [pdf].
